# Understanding the use of Flexible Work Arrangements Among Older New Zealand Caregivers

**DOI:** 10.1177/07334648231152153

**Published:** 2023-01-14

**Authors:** Shanika Yoshini Koreshi, Fiona Alpass

**Affiliations:** 1 6420 Massey University, Palmerston North, New Zealand

**Keywords:** older workers, caregiving, flexible work arrangements, employment

## Abstract

Flexibility in the workplace has been suggested to promote prolonged employment among older workers. This study focuses on the question of whether the use of flexible work arrangements (FWAs) differs between caregivers and non-caregivers and how potential differences can be explained. Participants were 296 carers and 1611 non-carers (aged 55–70 years) who completed the 2018 Health, Work and Retirement survey and were in paid employment. Hierarchical regression was used to investigate caregiving as an independent predictor of use of FWAs after controlling for demographic and work-related variables. Results show that caregivers on average used more FWAs than non-caregivers, including flexible work hours, flexible schedules and time off. Differences in FWAs use between caregivers and non-caregivers cannot be explained by differences in socio demographic and work characteristics. The use of FWAs warrants attention in discussions about prolonged employment and reconciliation of care and work among older adults.

**What this paper adds**

• Older adults who combine paid work and caregiving use more flexible work arrangements than older working adults who are non-caregivers.
• Older adults who combine paid work and caregiving are more likely to use three categories of flexible work arrangements; flexibility in the number of hours worked, flexible schedules and time off arrangements.
• Contributes to the limited literature on older workers reconciling paid work and informal caregiving.

**Applications of study findings**

• Organizations can design improved human resource policies to help older adults navigate the complexities of combining work and care.
• Contributes to the evidence base that informs government policy development on workplace policies for older adults who combine paid work and care.
• Informs policy to address barriers to women’s workforce participation due to caregiving responsibilities.

## Introduction

The rapid ageing of the population is driving an ageing workforce. This demographic shift will continue to result in an increasing number of older people in the labour force. Over the last decade, people aged 55 and above have had a significant growth in labour force participation rates (OECD, 2022). An ageing population also increases the need for long-term care due to the increasing prevalence of chronic health conditions (Etkind et al., 2017). Policy initiatives such as ageing in place have been proposed to encourage families to provide care for their loved ones in order to reduce the societal expense of providing residential care (Schofield et al., 2006). The increasing provision of informal caregiving (Pavolini & Ranci, 2008) and the extension of working lives (Davey, 2006) will result in many older workers combining paid work and informal caregiving responsibilities (Koreshi & Alpass, 2022).

Informal caregiving demands substantial effort, productive time and financial resources of caregivers (Brinda et al., 2014). Combining care with paid work may increase psychological distress and work overload for caregivers (Stephens et al., 2001), lead to poorer self-rated health (O’Loughlin et al., 2017), high caregiver burden (Gordon et al., 2012), and may result in caregivers opting for long-term sickness absence (Mortensen et al., 2017) or even employment exit (Carr et al., 2018). It is suggested that flexible work arrangements (FWAs) can help reduce work/family conflict and work interruptions and facilitate caregivers to combine their work and care responsibilities (Clancy et al., 2020).

Specific flexible work arrangement policies have been enacted in many countries to support working caregivers. In New Zealand, employees have the statutory ‘right to request’ flexible working arrangements from the first day of employment and employers have a duty to consider any requests under this right (MSD, 2009). However, this policy does not guarantee that the request will be granted as it is not mandatory for employers to consent to a request.

### What Flexible Work Arrangements do Caregivers Use?

Research on the use of flexible work arrangements by older adults to facilitate the combination of paid work and care is limited. A multidisciplinary prospective population-based cohort study in Netherlands explored the types of self-reported work arrangements (taking time off, formal care leave arrangements, individual agreements with the supervisor and a reduction in paid work hours) used by working caregivers aged 26–68 years (Oldenkamp et al., 2018). Just under a third (31%) of all caregivers had used at least one of these work arrangements while 16% had used at least two. Taking time off (41%) was the most common work arrangement utilized followed by individual agreements with a supervisor (30%). Data sourced from the 2015 ABS Survey of disability, Ageing and Carers (SDAC) in Australia was used to examine the availability, usage and barriers to accessing special work arrangements among caregivers and non-caregivers aged 15 years and above (Temple et al., 2019). Of the full sample, approximately 94% had access to at least one type of special work arrangement. Of the primary caregivers, 64% had used special work arrangements compared to 19% of non-caregivers. Paid leave and paid carers leave were the most often used arrangements. Of those who used arrangements, 15% wanted to use additional arrangements but, were unable to do so due to barriers such as not enough paid leave, or pressure from bosses/other workers. Caregivers were more likely to report barriers to further use of work arrangements than non-caregivers. Neither of these studies focused specifically on older adults combining work and care.

In a New Zealand study of older workers (aged 55+), nearly 60% considered flexible work schedules as important (Stephens et al., 2014). However, less than half of these participants reported that their employer offered these arrangements. Furthermore, only a quarter of working caregivers in the study had requested FWAs from their employer. Instead, many had used their annual leave, sick leave, or opted for leave without pay to undertake caregiving responsibilities (Stephens et al., 2014).

Limited studies address how caregiving factors and work characteristics play a role in the use of FWAs among older adults. Caregiving experiences are not homogenous (Cameron, 2021) and can differ in their level of care intensity (Broese Van Groenou et al., 2013). For instance, chronic ongoing conditions such as dementia will require different tasks and responsibilities compared to acute hospitalizations such as for a fall. Often, events that require immediate caregiving actions are unplanned and occur in unexpected crisis situations (Plaisier et al, 2015). These different experiences will necessitate different work arrangements in order to successfully reconcile work and care. The relationship between caregiver and care recipient may also drive the types of FWAs caregivers need and use. Research focused on adult-child caregivers (e.g., Olderkamp et al., 2018) may not be generalizable to spousal or other caregivers. Moreover, past studies do not account for self-employed caregivers (Oldenkamp et al, 2018). Self-employment can result in highly variable work patterns and provides a work structure that may be desirable to caregivers (Carmichael & Charles, 2003).

The way in which work flexibility has been operationalized in previous studies varies considerably (Allen et al., 2013) from a dichotomous ‘yes’ or ‘no’ to establish if a FWA is available and/or used (Oldenkamp et al, 2018), to the measurement of the degree of flexibility available in the workplace (Marche et al., 2020). Aggregating flexibility to a single variable may mask the differential effects of types of flexibility (Allen et al., 2013). For example, an employee might have the flexibility of working from home (flexiplace), but limited flexibility in scheduling the work (flexitime). In addition, limited studies distinguish between *access* to and *use* of FWAs (Allen et al., 2013; Chandola et al., 2019; Temple et al., 2019) when having access to FWAs does not always result in use (Allen et al., 2013).

### Present Study

Flexible work practices are considered to be an avenue to manage the demands of combining care and employment (Bainbridge & Broady, 2017). However, there is limited research to understand what type of FWAs are used by older caregivers. Given the rapidly ageing workforce and the growing need for older adults to combine work and care, it is important to understand how workplaces can facilitate arrangements to allow caregivers to remain in the labour force.

The present study will address previous limitations by incorporating a sample that includes *both* caregivers and non-caregivers above the age of 55 years; investigating the *use* of FWAs; accounting for caregiving and work characteristics that are associated with the use of FWAs; and finally, we will investigate the role of self-employment for older workers combining work and care. We examine the following research questions:

1. What type of FWAs are used by older caregivers versus non-caregivers?
2. Is caregiver status associated with the use of FWAs?
3. Does the use of FWAs differ based on caregiving characteristics among older caregivers?
4. Are self-employed caregivers more likely to use FWAs than employed caregivers?

## Methods

### Sample

The sample for the current study was drawn from the longitudinal Health, Work and Retirement (HWR) study established in 2006. This study employs a population level postal survey carried out biennially which aims to identify the health, economic and social factors underpinning successful ageing for people aged 55 years and over living in New Zealand. Participants are randomly selected from the New Zealand electoral roll, oversampling for persons indicating Maori descent to ensure adequate representation. The survey includes questions pertaining to health and wellbeing, work and retirement, social support and participation, income and financial wellbeing, and demographics. More information on the study and its methodology can be found elsewhere (Allen et al., 2019).

### Participants

Participants who responded to wave 8 (2018) were included in the present study. A total of 3965 returned completed surveys. Due to the nature of the study, the sample only included older adults who were in paid employment. Participants aged over 70 years were excluded from the final sample due to overall levels of workforce participation in this age group resulting in a final sample of 1907 older adults including 296 caregivers.

### Measures

#### Types of Flexible Work Arrangements

Participants were asked to review 18 flexible work arrangement (FWA) policies (adapted from Rudolph & Baltes, 2017), generally applicable to a variety of jobs, and indicate whether they had access to them or not. These work arrangements were grouped into 5 different categories based on the typology outlined in Pitt-Catsouphes et al. (2009): *Flexibility in number of work hours* (5 items) captures options provided by the employer for the number of hours one works in a given week, month or year, for example, ‘Input into the amount of overtime hours you work’; *Flexible schedule* (4 items), which captures work schedule options, for example, *‘*Frequently request changes in starting and quitting times’. *Flexible place* (2 items) indicates options provided with regard to the location of work, for example, ‘Work from an off-site location such as home for part or all of the regular work week’; *Options for time off* (4 items) comprised policies that allowed employees to take time off, for either short or extended periods of time, so that the employee could meet responsibilities at work and/or at home, for example, ‘Take paid leave for caregiving or other personal or family responsibilities’. *Other options* (2 items) did not fall into any of the above categories, for example, ‘Control when you take breaks’.

Each FWA was assessed using the following options ‘No, I do not have access to this’. ‘Yes, I have access to this, but I do not do this’. ‘Yes, I have access to this, and I do this’. The responses were recoded to obtain a dichotomous variable for usage. Use of each FWA policy was assigned a score of 1 (non-use = 0). These scores were summed for each item within each of the five FWA categories. A higher score indicated greater use of FWAs. The present study focuses on older workers who use FWAs, therefore, older workers who reported they did not have *access* to any FWAs were excluded (*n* = 127). The scale used in the current study does not account for individuals who do not have access to FWAs but may still use them. FWA access and use scores were highly correlated in the current sample [*r* (1,907) = .76, 95% confidence interval (CI) = .74, .78] indicating that use increased with access.

#### Caregiving Characteristics

Participants were asked whether they had provided care for someone with a long-term illness, disability, or frailty for at least 3 hours a week within the last 12 months; caregiver status was categorized as (yes = 1 and no = 0). Participants were also asked the following questions about their caregiving experience; number of hours of care provided weekly, care recipient’s living arrangement (living with carer, living alone, living in a nursing/caring facility, other).

#### Work Characteristics

To understand work characteristics of the participants, the following variables were used; the number of hours in paid employment per week; occupation (professional = 1 vs non-professional = 0); work status (full-time work = 1 part-time work = 0 (<30 hours/week)) and employment type (employed = 1 vs self-employed = 0).

#### Sociodemographic Variables

Gender (female = 1 vs male = 0), age (in years) and marital status (married/partnered = 1, single = 0) were measured. Socioeconomic status was measured using the Economic Living Standards Index (Jensen et al., 2005). This is a 25-item scale which measures participant’s financial and economic wellbeing. It is a multidimensional instrument that measures restrictions in social participation, restrictions in ownership of assets, economizing behaviour and self-reported standard of living. A total score can be derived by summing all the items with a range of 0 to 31. Scores can be used to categorize participants to ordinal groups ranging from severe hardship to very good economic living standards.

### Data Analytic Plan

Independent *t*-tests, χ^2^ and analysis of variance (ANOVA) were used to test group differences on sociodemographic factors, work-related factors and use of FWAs between the caregiver and non-caregiver groups. Hierarchical regression analyses were undertaken to examine whether caregiver status predicted the use of FWAs when controlling for sociodemographic and work-related factors. Pearson correlations and a Kruskal–Wallis H test were used to analyse the association and statistical differences between caregiving characteristics and the use of FWAs. Independent t-tests were used to determine statistical differences in the use of FWAs between self-employed and employed caregivers. All analyses were conducted using the Statistical Package for Social Sciences, SPPS (version 27.0).

## Results

Univariate comparisons indicated there was no difference between caregivers and non-caregivers on age, occupation, or employment type (see Table 1). However, significant differences emerged on gender, marital status, economic living standards, work status and work hours. Participants who identified as caregivers were more likely to be females, married, had lower scores on economic living standards, were employed in mostly part-time jobs and reported fewer hours of work than non-caregivers.

**Table 1.** Descriptive Statistics of predictor variable by caregiving status.

	Caregivers M (SD) % (*N* = 296)	Non-caregivers M (SD) % (*N* = 1907)	Significance
Age	61.67 (4.23)	62.04 (4.22)	t = −1.36, ns
Gender			
Male	30.4	48.1	X^2^ (1) = 31.4, *p* < .001
Females	69.6	51.9	Cramer’s V = .13
Marital status			
Married	70.4	78.7	X^2^ (1) = 9.53, *p* < .05
Single	29.6	21.3	Cramer’s V = .07
Economic living standards	23.6 (6.46)	25.4 (5.57)	t = −4.52, *p* < .05
Occupation			
Professional	28.8	23.9	X^2^ (1) = 2.80, ns
Non-professional	71.2	76.1	
Work status			
Full time	38.9	32.4	X^2^ (1) = 4.24, *p* < .05
Part-time	61.1	67.6	Cramer’s V = .05
Employment type			
Self-employed	20.9	22.4	X^2^ (1) = .29 ns
Employed	79.1	77.6	
Work hours	33.0 (15.3)	35.0 (14.8)	t = −2.04, *p* < .05

Significant differences between caregivers and non-caregivers in their use of FWAs were observed. Caregivers were more likely to use flexible hours, time off and flexible schedule policies than non-caregivers, while the latter used more of the ‘Other’ policies on average (see Table 2). The ‘other’ option category consisted of (i) control when one takes breaks; and (ii) transferring to a job with reduced responsibilities and reduced pay if needed.

**Table 2.** Use of Flexible Work Arrangements Among Caregivers and Non-caregivers.

	Caregivers *N* = 296	Non-caregivers *N* = 1515	P
Use of FWA			
Flexibility in no. of hours worked (range 0–5)	M = 1.05	M = .92	t(1809) = 1.84, *p* < 0.001 d = 0.11
Flexible schedule (range 0–5)	M = 1.30	M = 1.07	t(1809) = 2.94, *p* < 0.05; d = 0.18
Flexible place (range 0–2)	M = .28	M = .26	t(1809) = 0.62 ns
Time off (range 0–4)	M = .75	M = .64	t(1809) = 1.90, *p* < 0.05; d = 0.12
Other (range 0–2)	M = .58	M = .69	t(1809) = −3.41, *p* < 0.001; d = 0.20

### Multivariate Findings

Prior to conducing a hierarchical multiple regression, relevant assumptions were tested. An inspection of correlation coefficients and Tolerance/VIF values indicated that multicollinearity was not a concern. The data met the assumption of independent errors. Examination of the Mahalanobis distance scores indicated no multivariate outliers. Residual and scatter plots indicated the assumptions of linearity and homogeneity were all satisfied.

A series of two-stage hierarchical multiple regressions were conducted with the five FWAs as the dependent variables. The sociodemographic and work-related variables were entered at stage one. The caregiving variable (caregivers vs non-caregivers) was entered at stage two of the regression. The regression statistics are reported in table 3.

**Table 3.** Results of Regression Analyses to Explain Differences in Use of Flexible Work Arrangements, Coefficients and Standard Errors (*N* = 1999).

	Model 1: Flexible hours	Model 1A	Model 2: Flexible schedule	Model 2A	Model 3: Flexible place	Model 3A	Model 4: Time off	Model 4A	Model 5: Other	Model 5A
	Coefficient (SE)		Coefficient (SE)		Coefficient (SE)		Coefficient (SE)		Coefficient (SE)	
Age	.01 (.007)*	.01 (.007)*	−.02 (.007)**	−.02 (.007)**	−.002 (.003)	−.002 (.003)	−.01 (.005)	−.01 (.005)	−.01 (.003)*	−.01 (.003)
Gender^1^	−.20 (.06)**	−.22 (.06)**	−.14 (.07)**	−.16 (.070)**	−.10 (.03)**	−.10 (.03)**	−.08 (.05)	−.09 (.05)	−.14 (.03)**	−.13 (.03)**
ELSI	.02 (.05)**	.020 (.05)**	.02 (.01)**	.02 (.01)**	.01 (.002)**	.01 (.002)**	.02 (.004)**	.02 (.004)**	.02 (.002)**	.02 (.002)**
Marital status^1^	−.004 (.07)	−.01 (.07)	−.03 (.08)	−.04 (.07)	.003 (.03)	.002 (.03)	−.06 (.06)	−.06 (.06)	.04 (.03)	.04 (.03)
Work hours	.003 (.003)	.003 (.003)	.003 (.003)	.003 (.004)	−.003 (.001)	−.003 (.001)	.00 (.003)	.00 (.003)	.003 (.002)	.003 (.002)
Occupation ^1^	.03 (.06)	.04 (.06)	.09 (.07)	.11 (.07)	.10 (.03)**	.10 (.03)**	.09 (.05)	.08 (.05)	−.06 (.03)*	−.06 (.03)*
Work status ^1^	−.55 (.10)**	−.55 (.10)**	−.21 (.11)	−.21 (.11)	.04 (.05)	.04 (.05)	−.05 (.08)	−.04 (.08)	−.03 (.05)	−.03 (.05)
Employment status ^1^	−.28 (.07)**	−.28 (.07**	−.09 (.07)	−.08 (.07)	−.20 (.03)**	−.20 (.03)**	−.28 (.06)**	−.27 (.06)**	−.03 (.03)	−.03 (.03)
Caregiving status	—	.18 (.07)**		.28 (.08)**		.05(.03)		.15 (.06)**		−.07 (.04)
R^2^	.08	.09	.02	.03	.06	.06	.04	.04	.05	.06
Adjusted R^2^	.08	.08	.02	.02	.06	.06	.03	.04	.05	.05

Note * *p* < .05, ** *p* < .01.

Females = 1, Married = 1, Professional Jobs = 1, Full time = 1, and Employed = 1, Caregivers = 1.

### Flexible Hours

Age, gender, economic living standards, marital status, number of hours in paid employment per week, occupation, work status and employment status contributed significantly to the regression model (*F*(8, 1566) = 17.60, *p* < .001) and explained 8% of the variance in the use of flexibility in the number of hours worked (Table 3, Model 1). The caregiver status variable explained an additional .4% of the total variance and this change in *R*^2^ was significant, *F*(15, 1565) = 16.40, *p* < .001 (Table 3 Model 1A).

### Flexible Schedule

Age, gender, economic living standards, marital status, number of hours in paid employment per week, occupation, work status and employment status contributed significantly to the regression model (*F*(8, 1566) = 4.17 0, *p* < .001) and explained 2% of the variance in the use of flexible schedules (Model 2). The caregiver status variable explained an additional .7% of the total variance and this change in *R*^2^ was significant, *F*(15, 1565) = 5.03, *p* < .001 (Model 2A).

### Flexible Place

Age, gender, economic living standards, marital status, number of hours in paid employment per week, occupation, work status and employment status contributed significantly to the regression model (*F*(8, 1566) = 12.40 0, *p* < .001) and explained 6% of the variance in the use of flexible place arrangements (Model 3). Caregiver status was not a statistically significant contributor to the model *F*(15, 1565) = 11.25, *p* = .16 (Model 3A).

### Flexible Time Off

Age, gender, economic living standards, marital status, number of hours in paid employment per week, occupation, work status and employment status contributed significantly to the regression model (*F*(8, 1566) = 7.80 0, *p* < .001) and explained 3.3% of the variance in the use of flexible schedules (Model 4). The caregiver status variable explained an additional .4% of the total variance and this change in *R*^2^ was significant, *F*(15, 1565) = 7.643, *p* < .001 (Model 4A).

### Flexible Other

Age, gender, economic living standards, marital status, number of hours in paid employment per week, occupation, work status and employment status contributed significantly to the regression model (*F*(8, 1566) = 11.19, *p* < .001) and explained 5% of the variance in the use of other flexible arrangements (Model 5). Caregiver status was not a statistically significant contributor to the model *F*(15, 1565) = 11.25, *p* = .07 (Model 5A).

### Caregiving Characteristics and Use of Flexible Work Arrangements

Pearson product-moment correlations were run to examine the relationships between the five categories of FWAs and caregiving characteristics. There were no significant relationships between caregiving hours and any of the five flexible work arrangement categories (see Table 4).

**Table 4.** Results of Correlation Analyses to Explain Associations Between Study Flexible Work Arrangements and Hours of Caregiving Provided per Week (*N* = 249).

Variables	*M* (*SD*)	1	2	3	4	5	6
1. Caregiving hours	20.04 (2.18)	—	0.02	−0.09	0.05	−0.01	−0.05
2. Flexibility in no. of hours worked	0.92 (0.03)		—				
3. Flexible schedule	1.08 (0.03)			—			
4. Flexible place	0.26 (0.01)				—		
5. Time off	0.65 (0.02)					—	
6. Other	0.66 (0.01)						—

There were also no statistically significant differences between the care recipient’s living arrangement and the five FWAs (see Table 5).

**Table 5.** Means, Standard Deviations and One-Way Analyses of Variance of Flexible Work Arrangements by Care Receiver’s Living Arrangements (*N* = 212).

	Living with carer	Living alone	Living in nursing/care facility	Other	*F (1,212)*
	*M* (*SD*)	*M* (*SD*)	*M* (*SD*)	*M* (*SD*)	
Flexibility in no. of hours worked	.73 (.95)	.50(.52)	.73 (1.27)	1.15 (1.32)	2.41
Flexible schedule	1.33(1.38)	1.17(1.12)	1.27(1.01)	1.32(1.31)	.06
Flexible place	.31 (.55)	.25(.45)	.00(.00)	.34 (.58)	1.34
Time off	.82 (.86)	.25(.45)	.36(.67)	.84(1.06)	2.04
Other	.53(.54)	.58(.52)	.64(.51)	.61(.60)	.26

### Employment Type and Use of Flexible Work Arrangements

Inspection of Q-Q plots revelated that the five types of FWAs were normally distributed for both self-employed and employed caregivers. An independent t-test was conducted to compare the mean FWA category scores for self-employed and employed caregivers. The use of flexibility in number of hours worked t (264) = 3.92, *p* = .02, flexible place t (264) = 3.95, *p* = .001, time off t(264) = 2.48, *p* = .005 and other arrangements t(264) = 3.34, *p* = .002 in the self-employed group was significantly higher than the employed group. The use of flexible schedules was not significantly different among self-employed and employed groups t (264) = 1.54, *p* = .23.

## Discussion

An ageing workforce and the need for prolonged labour force participation is a challenge for older workers who combine both paid work and caregiving. Flexibility in the workplace has been suggested as a valuable support system to enable continued employment for those providing informal family care. This study focused on understanding the use of such flexible arrangements by older working caregivers and whether use differed to their non-caregiver counterparts.

### Type of Flexible Work Arrangements Used by Older Caregivers versus Non-caregivers

We found that more than ninety percent (93.8%) of older workers who were also providing informal care used one or more FWAs compared to 85% of non-caregiving older workers. Of the five FWAs categories, caregivers and non-caregivers significantly differed on the use of three. Caregivers were more likely to use the time off category (i.e. paid leave for caregiving and family responsibilities, unpaid vacation days or temporary career breaks) compared to non-caregivers. Additionally, caregivers were more likely to use categories of FWAs that allowed flexibility in the number of hours worked and flexible work schedules. Opting for flexibility in the number of hours worked, flexible schedules and time off provides carers with more control to manage work and caregiving (Lero & Fast, 2018), particularly those with episodic caring responsibilities. These types of arrangements provide autonomy to plan and schedule responsibilities with fewer interruptions. In comparison, flexible place arrangements such as working from home where the care recipient may also reside, could lead to more work interruptions. For instance, carers may feel distracted or feel guilty that they cannot pay more focused attention to the care recipient (Spann et al., 2020).

The two groups differed on socioeconomic and work-related factors, and these have also been shown to contribute to the use of FWAs. For instance, previous research has found older women report less perceived flexibility in their working hours, work schedules and workplace than older males (Damman & Henkens, 2020); and requests by higher skilled workers for FWAs may be more readily accepted by employers (Brescoll et al., 2013). In the present study, caregivers were more likely to be female, married, of lower socioeconomic status and work part-time jobs with fewer hours. However, when controlling for these factors, caregiving status still contributed to the use of three FWA categories: flexible hours, flexible schedule and time off, suggesting that there are characteristics associated with the caregiving role itself that may influence use of FWAs. Other organizational policies favourable to caregivers (apart from FWAs) which may influence where caregivers choose to work, may also partially account these differences.

### Flexible Work Arrangements and Caregiving Characteristics Among Older Caregivers

Past research shows that characteristics such as caregiver burden, caregiving intensity and severity of care recipient’s health are associated with the need for FWAs (Plaisier et al., 2015). As noted by Oldkenhamp et al. (2018), such studies examined perceived need and not use of FWAs. In the present study, which focused on *use* of FWAs, caregiving characteristics such as number of hours of care provided and care recipient’s living arrangements were not associated with the use of any of the categories of FWAs. These findings may reflect the way that caregiver characteristics have been operationalized in the current research which measured caregiver demand rather than burden. Oldkenhamp et al. (2018) suggest that unlike caregiver burden (i.e. the degree to which caregiving interrupts daily activities and physical health), caregiver demands do not fully capture how caregivers experience their caregiving. The lack of any significant relationships between caregiving characteristics and use of FWAs should be interpreted with caution given the relatively small sample of caregivers.

### Employment Type and Use of Flexible Work Arrangements

Previous FWA research on caregivers has lacked a focus on the role of self-employment, a work status that may be conducive to caregiving demands (Carmichael & Charles, 2003). In the present study, self-employed caregivers were more likely to use flexibility in number of hours, flexible place, time off and other flexible arrangements than employed caregivers. The number of self-employed in the 50+ age group is growing in New Zealand (Pearman et al., 2022; Statistics NZ, 2021). Clearly, self-employed caregivers have more flexibility to arrange their work hours (Carmichael & Charles, 2003), a potential mechanism for managing work-life balance for caregivers (Bourke, 2009), particularly in later work life.

### Limitations

Due to the small caregiver sample in the current study, we were unable to investigate possible interactions between caregiving status and other explanatory variables (i.e. gender and employment status) on the use of FWAs. For instance, the caregiver literature has consistently shown that females are more likely to take up caregiving responsibilities than males (Dentinger & Clarkberg, 2002). In addition, women have different work histories to men suggesting that gender may impact on older workers opportunities to access FWAs (e.g., seniority, skill level) (Damman & Henkens, 2020). Also, in our study population, 20.1% of caregivers reported being self-employed, similar to that in the general population in this age group (Statistics NZ, 2021). Investigating these possible interactions will inform how policies and practices around FWAs should be tailored to support older working adults who provide care.

The present study focused on use of FWAs rather than access. Based on the assumption that participants who do not have access to FWAs do not have the ability to use them, those who reported they had no access to FWAs were excluded from the sample. In doing so, we may have introduced a selection bias in that caregivers may be self-selecting into those jobs that provide access to FWAs. Post-hoc analyses on access to FWAs revealed that only access to the ‘Other’ FWA category differed between caregivers and non-caregivers, with caregivers slightly more likely to say they had access to this FWA compared to non-caregivers. Overall, our findings suggest that both groups are as likely to choose jobs with access to FWAs, however, caregivers are more likely to use them.

It should also be noted that only those older caregivers who have been successful in combining work and care are included in our sample (survivor bias). Those who have exited employment due to caregiving responsibilities may have done so due to different, less accommodating, patterns of access and use.

The reference period for providing care and employment is not congruently captured in the HWR survey. We acknowledge that a small number of these participants who provided care in the last 12 months and are currently working may not necessarily be currently providing care. Caregivers in our study provided a minimum of 3 hours of care for someone with a long-term illness, disability or frailty within the last 12 months. There were 395 participants who had worked less than a year for the current employer and 14 of these participants also identified as caregivers.

Finally, consideration should be given to the possibility of reverse causation in that having access to and use of FWAs may persuade and assist older workers to take on a caring role.

Despite the limitations, our study has several strengths. By utilizing an older adult sample, we are addressing a fast-growing section of the labour force and an under studied demographic who are increasingly combing work and care. We have contributed to a research gap by investigating the use of FWAs among caregivers as opposed to only evaluating the perceived need or access to FWAs. Having access to FWAs does not necessarily determine usage, thus, it is important to distinguish use from availability (Allen et al, 2013). We also examined the role of self-employment as this employment status provides caregivers with potential opportunities for flexibility, particularly for older workers.

### Future Research

This research was undertaken before the covid pandemic which had a major impact on the use of flexible work arrangements, in particular, place of work. Recent research indicates that working remotely can lead to both positive and negative impacts on productivity (Vyas, 2022). Some workers prefer the flexibility to manage work time and place, whereas others experience interruptions by family members that affect productivity when working from home. While most countries have now reverted to pre-pandemic regulations around isolation and movement, whether pre-pandemic findings still hold in the ‘post-pandemic’ environment requires further study.

Future research should investigate the relationship between age and preferences for flexible work arrangements. This is particularly salient for older workers as they have different work needs than younger workers and are susceptible to more workplace barriers (Harris et al., 2018).

Our findings show that older working caregivers use more FWAs than non-caregivers across three FWA categories. Future research should aim to understand if and how these FWAs support older adults to reconcile paid work and care responsibilities.

## Conclusion

This paper sought to understand the use of FWA among older workers with a focus on those with caregiving responsibilities. The evidence from this study suggests that caregivers use more FWAs than non-caregivers when controlling for work and sociodemographic factors. While providing some initial indications of the types of FWAs used, future studies should investigate the effectiveness of different FWAs in addressing the differing needs of working carers in order to develop organizational policies to support an ageing workforce with increasing caregiving responsibilities.
